# Review on Natural Preservatives for Extending Fish Shelf Life

**DOI:** 10.3390/foods8100490

**Published:** 2019-10-13

**Authors:** Jun Mei, Xuan Ma, Jing Xie

**Affiliations:** 1 College of Food Science and Technology, Shanghai Ocean University, Shanghai 201306, China; 2 National Experimental Teaching Demonstration Center for Food Science and Engineering Shanghai Ocean University, Shanghai 201306, China; 3 Shanghai Engineering Research Center of Aquatic Product Processing and Preservation, Shanghai 201306, China; 4 Shanghai Professional Technology Service Platform on Cold Chain Equipment Performance and Energy Saving Evaluation, Shanghai 201306, China

**Keywords:** natural preservatives, fish, spoilage mechanisms, application

## Abstract

Fish is extremely perishable as a result of rapid microbial growth naturally present in fish or from contamination. Synthetic preservatives are widely used in fish storage to extend shelf life and maintain quality and safety. However, consumer preferences for natural preservatives and concerns about the safety of synthetic preservatives have prompted the food industry to search natural preservatives. Natural preservatives from microorganisms, plants, and animals have been shown potential in replacing the chemical antimicrobials. Bacteriocins and organic acids from bacteria showed good antimicrobial activities against spoilage bacteria. Plant-derived antimicrobials could prolong fish shelf life and decrease lipid oxidation. Animal-derived antimicrobials also have good antimicrobial activities; however, their allergen risk should be paid attention. Moreover, some algae and mushroom species can also provide a potential source of new natural preservatives. Obviously, the natural preservatives could perform better in fish storage by combining with other hurdles such as non-thermal sterilization processing, modified atmosphere packaging, edible films and coatings.

## 1. Introduction

Fish has high protein content and low saturated fat content, which is considered as highly valuable food [1]. In particular, fish is the primary dietary source of omega-3 polyunsaturated fatty acid (PUFA), including docosahexaenoic acid (DHA) and eicosapentaenoic acid (EPA), both of which are well-known for the anti-inflammatory action and protective effects on cardiovascular disease [2,3,4]. The World Health Organization (WHO) recommends a regular fish consumption of 1–2 servings per week to provide the equivalent of 200–500 mg of omega-3 PUFA [5,6]. According to statistics, fresh and live fish account for about half of the total seafood consumed by human beings. The word “fresh” refers to fish that have not been frozen, including still alive fish as well as kept in the cold but not frozen section or packed in modified atmosphere [7]. Unlike frozen fish, the fresh fish cannot stay as inventory for one month [8]. For consumers, freshness is also often associated with safety, reassurance and superior taste. In most cases, the customers believe that the fish sold in market in China are caught recently. However, it could take more than a week to arrive at these stores. Consumers are still unsure whether the “fresh” product is really fresh or has been frozen and then thawed [9,10]. 

Fresh fish can easily deteriorate after being captured due to the endogenous enzyme and rapid microbial growth naturally present in fish or from contamination [11]. In the process of fish decay, decomposition of various components and formation of new compounds occur. What is more, changes in composition during fish decay lead to protein degradation and lipid oxidation, as well as changes in fish odor, flavor, and texture. Therefore, it has become inevitable to develop effective treatment methods to extend the shelf life of fish [12]. Soft or mushy texture of fish limits the shelf life, thereby impeding its marketing. During postmortem handling and storage, the holding temperature, oxygen, endogenous or microbial proteases, moisture can result in detrimental changes in the color, odor, texture, and flavor of fish [13,14]. Therefore, fish have traditionally been cooled and stored in flake ice, refrigerated sea water, or ice slurries or they have been preserved by exposure to chemical agents. At the same time, the fishery industry is always looking for new preservation methods to extend the fish shelf life and provide consumers with the best quality in terms of sensory and nutritional levels [15].

In recent years, researchers have put much effort into searching natural preservatives that could inhibit the growth of bacteria and fungi in food. Meanwhile, a growing number of consumers are aware of the potential negative health effects of chemical preservatives, which has prompted the food industry to find natural products used and developed as alternatives. Natural preservatives are available from a variety of sources including plants, animals, bacteria, algae, and fungi [16,17]. Microbial derived preservatives (e.g., bacteriocin), plant derived preservatives (thyme essential oil, tea polyphenols, rosemary extract, etc.), and animal derived preservatives (e.g., chitosan from crab or shrimp shells) have been demonstrated to have antimicrobial or antioxidant properties. In addition, antimicrobial compounds produced by algae and fungal (mushroom) could be served as potential sources of new antimicrobial substances for use as natural preservatives in food. The aims of this review were to expound the factors affecting the quality of fish by introducing the corruption mechanisms of these products and summarize natural preservatives derived from microbial-, plant-, animal-derived compounds, algae and mushrooms. 

## 2. Spoilage Mechanisms

Fish is extremely perishable compared to other muscle foods and will enter into rigor mortis where fish lose their flexibility due to the stiffening of their muscle just a few hours after death [18]. Studies show that the spoilage of fish results from three basic factors: enzymatic autolysis, oxidation, and microbial growth [19]. 

### 2.1. Autolytic Enzymatic Spoilage

As the degradation process of fish begins with autolytic enzymatic spoilage, chemical and biological changes take place in dead fish shortly after capture due to autolytic enzymatic breakdown of major molecules. The autolytic enzymes had a significant impact on textural deterioration (softening) and therefore on spoilage; however, they did not produce the characteristic spoilage off-flavors and off-odors [20,21]. Some studies have demonstrated that the fish quality could deteriorate even though comparatively low levels of autolytic degradation were present and limit the shelf life of fish [22,23]. 

Some gastrointestinal digestion enzymes as well as endogenous muscular enzymes are found in the viscera and muscle of fish after being caught. These enzymes can be conducive to postmortem degradation in fish muscle and fish products during processing and storage, which can lead to a sensory or product associated change. On the other hand, the autolysis of fish muscle proteins could also result in fish meat spoilage and biogenic amines production. The degree of fish freshness has to be determined in the autolysis stage before the spoilage of fish begins [21]. It has been pointed out that low temperature and a_w_ kept during the storage period can maintain a low activity of the endogenous autolytic enzymes in muscle [24]. In addition, the temperature of vacuum-packed cold-smoked salmon storage should be at low temperature to maintain low native enzyme activity in the tissue [25].

### 2.2. Oxidative Spoilage

Lipid oxidation could result in spoilage and deterioration and contained a three-stage free radical mechanism: initiation, propagation, and termination [26,27,28]. Some studies suggested that lipid oxidation in fish may be initiated and propagated by a number of mechanisms including auto-oxidation, lipoxygenase, microsomal enzymes, photosensitized oxidation, and peroxidase [29]. In addition, iron-bound proteins in fish muscles, such as hemoglobin, ferritin, myoglobin, hemosiderin, and transferrin, as well as other metals, may be released during storage and play an important role in initiating and/or activating lipid oxidation [30]. 

Lipid oxidation can be divided into enzymatic and non-enzymatic oxidation, both of which can result in serious decreases in fish qualities. It is typically recognized that oxidation involves the reaction of oxygen with the double bonds of fatty acids. Fish is rich in unsaturated fatty acids and prooxidant molecules, which are extremely susceptible to oxidation resulting in rancidity development and quality loss [31]. These compounds result in deteriorations of smell, color, texture, and nutritional values. Lipid oxidation products also have been demonstrated to promote protein denaturation, modification of protein electrophoretic profiles, nutritional losses, endogenous antioxidant systems losses, and developments of fluorescent compounds [32]. Accordingly, lipid oxidation leads to a decrease of fish acceptability by consumers [33].

### 2.3. Microbial Spoilage

Fish has high contents of free amino acids, a high post mortem pH, high water contents, and many fish species contain trimethylamine oxide (TMAO), which promote bacterial growth in a wide temperature range [34]. The microbial growth is considered to be the major cause of the deterioration of fish quality, causing up to 25–30% loss of such products [17]. It is generally believed that each fish has its unique flora, which is determined by raw materials, processing parameters, subsequent storage conditions, and microbial tolerance to storage conditions [35]. For example, it was reported that spoilage microorganisms for aerobically stored frozen fish including salmon were species within the genera *Shewanella* (*S.*) and *Pseudomonas* (*P.*), while the CO_2_-resistant *Photobacterium (Ph.) phosphoreum* dominated on fish under modified atmosphere packaging [34,35,36]. 

It is important to distinguish non-spoilage bacteria from spoilage ones as many of the bacteria present do not actually contribute to spoilage during fish storage [37]. A variety of psychrotrophic Gram-negative bacteria comprise the main part of the initial microbiota of fish from temperate sea waters. However, only a small fraction of fish microbiota is responsible for spoilage, known as specific spoilage organisms (SSOs). The SSOs could dominate and produce the metabolites through assimilation of nonprotein-nitrogen of fish muscle with unpleasant and unacceptable off-flavors, which directly affect the organoleptic properties of fish resulting in its rejection by the consumers [38]. The SSOs were different for different fish species and preservation conditions. Research studies demonstrated that *Pseudomonas* spp. was the SSOs for Atlantic salmon (*Salmo salar*) in modified atmosphere packaging and bighead carp (*Aristichthys nobilis*) with 2% salt, whereas *Aeromonas* was the SSOs for unsalted bighead carp [39].

## 3. Natural Preservatives for Fish 

Fresh fish is highly perishable, and even if it can be refrigerated or frozen to extend its shelf life, these processes may not be sufficient to prevent lipid oxidation, rancidity or bacterial growth. In most cases, it is also necessary to improve the quality of fish [40]. For this reason, it is required that preservatives should be properly added to fish during storage.

With the rapid development of social economy, the application of natural preservatives in food has attracted more and more attention from the public. Generally, the public will choose a food with no preservatives, but if these are not available, the same consumer will choose a food containing natural preservatives over synthetic ones [41]. Natural preservatives guarantee that the food is free of microorganisms and safe to eat. Ideally, natural preservatives should have broad bactericidal and fungicidal activities, be non-toxic, be active at low concentrations, impart no flavor or color to food, have no pharmaceutical applications, label friendly, and finally cost effective [42]. Natural preservatives generally come from three sources: microorganisms, animals, and plants. In addition, various bio-active compounds extracted from algae, mushrooms and so on can also provide a potential source of new natural preservatives in the food industry [43].

### 3.1. Microbial-Derived Compounds

Specific strains of lactic acid bacteria (LAB) produce some inhibitory substances (such as diacetyl, reutericyclin), antifungal compounds (such as phenyl-lactate, propionate, cyclic dipeptides, hydroxyphenyl-lactate, and 3-hydroxy fatty acid), bacteriocins, and bacteriocin-like inhibitory substances, which have antibacterial activity and can be exploited against spoilage bacteria and food-borne pathogens in fish during storage [44]. Some applications of microbial-derived compounds in fish preservation are depicted in Table 1. 

#### 3.1.1. Bacteriocins

Some studies have demonstrated that bacteriocins generally produced by LAB could be active against other relatively closely-related bacteria to obtain a competitive advantage of nutrients in the environment [45]. Bacteriocins are a group of potent antimicrobial peptides containing about 30–60 amino acids, forming amphiphilic helices, which differ in activity spectrum, mode of action, biochemical characteristics, molecular weight, and genetic origin. Bacteriocin-producing LAB strains could protect themselves from their own toxins by expressing a specific immunity protein, encoded in the bacteriocin operon [46,47]. It is generally recognized that bacteriocins can be categorized into four classes: I): lantibiotics, low molecular weight (<5 kDa) thermostable peptides, characterized by the presence of lanthionine and derivatives; II): small thermostable peptides (<10 kDa) composed of three subclasses: IIa (pediocin and enterocini), IIb (lactocin G), and IIc (lactocin B); III): high molecular weight (>30 kDa) thermolabile peptides, represented by helveticin J; and IV): large peptides complexed with lipids or carbohydrates [48,49,50].

Nisin was discovered by Rogers and Whittier in 1928, and was produced by certain strains of *L. lactis* subsp. Nisin structurally is a 34-amino acid polypeptide with a molar mass of 3500 Da and belongs to the lantibiotic class as containing methyllanthionine and lanthionine groups. Nisin has been industrially produced for specific applications to prevent spore germination and pathogen growth on the surface of contaminated food. It has been accepted for commercial application as a Generally Regarded as Safe (GRAS) food preservative by around 50 countries [51,52]. Nisin Z has higher solubility and diffusion characteristics, which plays an important role in the preservation of fish [53]. According to Sofra et al., nisin (2 × 10^4^ IU/100 g) in the osmotic solution delayed tuna slices spoilage and extended shelf life to 51 days at 5 °C [54]. Nisin at 1000 IU/g obviously decreased the *Listeria* population in the storage days [55]. Besides, nisin had a positive effect on color stability of rainbow trout during storage as the formation of hydrophobic bonds between carotenoids and the apolar fraction of nisin [51]. Nisin exhibits good antimicrobial activities against a wide range of Gram-positive bacteria and it may be useful against some Gram-negative bacteria together with other preservatives for fish storage. Nisin combined with chitosan treatment was a promising approach to maintain quality for large or small yellow croaker [56,57]. Some studies also showed that nisin combined with natural antioxidant (such as rosemary extract, essential oil or tea polyphenols) could effectively maintain or improve the sensory attributes, inhibit the microbial growth, delay the chemical changes in the fresh fish during storage and may be a promising method to maintain the storage quality [58,59,60,61]. In addition, nisin with some non-thermal processing can also extend the shelf life of fish, such as irradiation, vacuum packaged, and so on [62,63,64].

Pediocins, belonging to Class IIa bacteriocins, are small, cationic proteins with anti-*Listeria* activity. They share a highly conserved charged and hydrophilic charged N-terminal part containing the consensus sequence -YGNGV- and a more variable hydrophobic and/or amphiphilic C-terminal part. Pediocins exhibit important technological properties, such as maintaining activity over a wide pH range and thermostability, as well as antimicrobial effect on Gram-positive food spoilage and pathogenic bacteria, making them as an important class of biopreservatives for fish [65,66]. Yin et al. investigated the biopreservative effectiveness of pediocin ACCEL on refrigerated fresh fish fillets. Pediocin ACCEL was more effective than nisin on the suppression of *Listeria monocytogenes* growth in refrigerated fish during refrigeration [67]. It is typically known that occurrence and growth of *L. monocytogenes* in ready-to-eat fish remains a challenge, and *Listeria* outbreaks have previously been reported due to fish contamination [68]. These studies demonstrated that *Pediococcus acidilactici* ALP57, isolated from non-fermented shellfish (oyster, mussels, clams), could synthesize pediocin bac ALP57 (approximately 6.5 kDa, 12,800 AU/mL) that has the antimicrobial activity against *L. monocytogenes* ESB54 during its exponential growth phase [69]. 

Lacticin is another bacteriocin produced by *L. lactis* subsp. *Lactis* is a two-peptide bacteriocin possessing potent activity against Gram-positive bacteria [70]. However, it is worth noting that lacticin differs from nisin in its target specificity and greater effectiveness. In addition, the mechanism of action contrasts from the single nisin peptide as requiring the interaction of two peptides for optimal bactericidal activity [71]. Kim et al. showed that the lacticin NK24 could slow down the microbial growth on packaged fresh oysters and maintain the chemical quality and extended shelf life significantly as well [72].

#### 3.1.2. Reuterin

Reuterin is considered as a D-ribose analogue and is an intermediate compound generated by *Lactobacillus reuteri* during glycerol metabolism. It inhibits the substrate binding subunit B1 of ribonucleotide reductase, which catalyzes the reduction of ribonucleotides to their corresponding deoxyribonucleotides to inhibit DNA synthesis [73,74]. Due to its chemical properties and its antibacterial activity against food-borne pathogens and spoilage bacteria, reuterin has high potential as a food preservative [75]. As reported by Montiel et al., purified reuterin (10 AU/g) significantly inhibited *L. monocytogenes* growth in cold-smoked salmon kept under moderate or strong temperature abuse conditions [76]. Furthermore, reuterin together with high hydrostatic pressure at 450 MPa for 5 min also improved the safety and extend the shelf life of cold-smoked salmon [77]. Some studies have demonstrated that reuterin has higher antimicrobial activity against Gram-negative bacteria than that of Gram-positive ones [43]. Although the antimicrobial mechanism has not yet been fully understood, so far, reuterin is still considered to be a highly promising food bio-preservative.

#### 3.1.3. Organic Acids

Organic acids are organic compounds with one or more carboxyl groups (-COOH) in their structure and are known to possess antimicrobial properties in food application and have been studied extensively, and they are categorized as GRAS [87]. Application of organic acids on fish surfaces, mainly through dipping or spraying, is a widely-used and well-known practice. A range of experiments have shown that organic acids and their respective salts could inhibit bacterial growth in different varieties of fish. For example, García-Soto et al. suggested that the presence of lactic (0.50 g/L) and citric (1.25 g/L) acids in the ice medium led to a deteriorative activity inhibition and improve the quality of megrim and hake during the on-board chilled storage [88]. A novel citric, ascorbic, and lactic acid (at 800 mg/kg, respectively) icing system were proved to be an effective mixture for hake, megrim, and angler preservation due to its antimicrobial effect [15]. However, the direct addition of citric acid may negatively affect the texture and other sensory attributes of fish patties. Encapsulated citric acid could minimize secondary oxidation values regardless of the concentration, which represents an appropriate strategy in the fish preservation [89]. Besides, sodium salts of the low molecular weight organic acids, such as acetic, citric, and lactic acids, have also been used to control microorganism growth, improve sensory properties, and extend the shelf life of fish. The combination of citric acid and potassium sorbate was sufficiently effective in inhibiting microorganism growth and maintaining volatile base nitrogen at low levels for the fish preservation [90]. Sallam concluded that dipping of salmon slices in aqueous solutions (2.5%) of sodium lactate, sodium acetate, and sodium citrate could effectively inhibit the proliferation of spoilage microorganisms to delay lipid oxidation and extend the shelf life during cold storage [91]. Organic acids and salt have pleasant taste without being too tough, which can be employed for the marinated fish preservation. Frozen fillets of sardine were used to make marinades in 14% sodium chloride and 7% acetic acid in barrels for 22 days at 4 °C [92]. However, when fillets of sardine immersed in the marination solutions containing 10% sodium chloride and 2% or 4% acetic acid for 24 h, the trimethylamine (TMA-N) and total volatile base nitrogen (TVB-N) values significantly increased during storage [93].

### 3.2. Plant-Derived Compounds

#### 3.2.1. Essential Oils (EOs)

EOs are complex mixtures of volatile organic compounds (VOCs) produced as secondary metabolites in plants and frequently responsible for the characteristic odor of plants [94]. They are characterized by two or three major VOCs at fairly high concentrations (20–70%) compared to other VOCs [95]. Some EOs have antimicrobial and antioxidant properties and an increasing demand for natural preservatives has led to EOs as potential alternatives for antimicrobials and antioxidants [96]. EOs have been proved to be effective antimicrobials against some foodborne pathogens including *S. Typhimurium*, *E*. *coli* O157: H7, *Campylobacter, L*. *monocytogenes*, *S*. *aureus,* and others. Studies show that the efficacy of EOs depends on chemical structure, concentration, matching the antimicrobial activity spectrum with the target microorganism(s), interactions with the food matrix, and application method [97]. 

**Figure 1:**
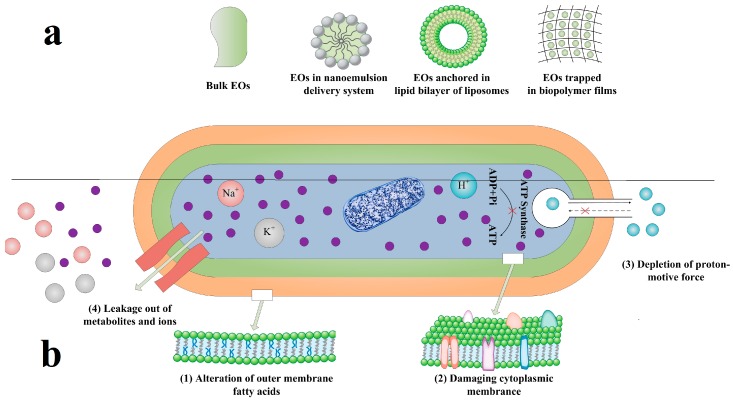
(**a**) Bulk EOs and different types of EO delivery systems, including nanoemulsion, liposomes, and biopolymer films; (**b**) Proposed common mechanisms of action and target sites of essential oils (EOs) or EO delivery systems on bacterial cell [110].

It has been observed that some EOs show inhibitory effect on membrane integrity against the tested food-borne pathogenic bacteria [98,99,100,101] (Figure 1). Intracellular material leakage is a general phenomenon results in cell death. The hydrophobic nature of EOs could interfere with bacterial lipid membrane resulting in increased permeability of the cell constituents [102,103], which is in agreement with other phenolic compounds [104,105,106,107]. So far, most studies concerning the antimicrobial action mode of EOs have been carried out on bacteria, while less is known about their effects on molds and yeast. Gram-positive bacteria are generally more susceptible than Gram-negative ones [108]. The cell wall lipopolysaccharides (LPS) of Gram-negative bacteria can create a barrier toward macromolecules and hydrophobic compounds, preventing active compounds in EOs reaching to cytoplasmic membrane [109]. The combinations of EOs with other natural preservatives or even other chemical ones also show positive effects. 

#### 3.2.2. Plant Extracts

Plant extracts have broad application prospects in fish preservation. The antimicrobial activities of plant extracts may be attributed to the combined effects of polyphenols adsorption to bacterial membrane with membrane disruption and subsequent cellular contents leakage, and the generation of hydroperoxides from polyphenols [111,112,113,114]. Plant extracts also show antifungal activities, antioxidant, antimutagenic activities, and inhibit lipid oxidation in food [115,116,117,118]. Numerous studies have been done in-vitro to evaluate the antimicrobial activities of plant extracts; however, only a few studies are available for fish preservation as the antimicrobial activity of plant extracts does not produce as marked inhibition as many of the chemical preservatives in fish. The plant crude extracts generally contain flavonoids in the form of glycosides, in which the sugar presenting in them decreases the effectiveness against some food-borne pathogens [119,120]. 

The antimicrobial and antioxidant effects of various plant-derived compounds on fish have been performed and summarized in Table 2. The plant-derived compounds could extend fish shelf life by reducing the total aerobic plate count and retarding lipid oxidation and may also be used together with other natural preservatives or different packaging ways [120]. Rainbow trout (*Oncorhynchus mykis*s) using turmeric extract, shallot extract, and their combination with vacuum packaging could reduce the growth of total viable count and extend the shelf life [121]. The plant-derived compounds are also combined with nisin to extend the shelf life of fish and fish products [55,61,122].

#### 3.2.3. Natural Wood Smoke 

Natural wood smoke is a suspension of vapors, liquid droplets, and solid particles and produced by controlled wood smoldering without oxygen or at reduced oxygen levels. Different woods’ smoke have different antimicrobial properties as the woods generate different levels of antimicrobials, such as organic acids, phenols, and carbonyls during pyrolysis. Now, more than 20 different kinds natural wood smoke including redwood, black walnut, hickory, birch, white oak, aspen, chestnut, and cherry have been evaluated the antimicrobial properties against *A. hydrophila,* and *S. aureus* [123]. Additionally, the smoke treatment could increase redness of the fish muscle and stabilized it during frozen storage [124]. It should be noted that wood smoke contains some harmful compounds, such as polycyclic aromatic hydrocarbons (PAHs) [125,126]. Since the 1970s, liquid smoke has been developed and become popular resulting from the concern of potentially carcinogenic benzopyrenes [127]. Liquid smoke preparations can be either incorporated as a surface additive during post thermal processing or a formula ingredient during batter mixing to reduce or eliminate food-borne pathogens as well as impart desired smoky flavor to the products. Many studies have been focused on the use of liquid smoke as a postlethality dip or spray treatment to reduce or eliminate food-borne pathogens on fish products. Antimicrobial efficacy of liquid smoke can be enhanced by vacuum-packaging, essential oils, and NaCl [128,129,130,131,132]. Also, wood smoke can be converted into nanocapsules using chitosan and surface contact area could be increased, which delayed microbial growth in fish fillets at cold storage conditions [133,134].

#### 3.2.4. Algae and Mushrooms

As natural sources of bioactive compounds, algae and mushrooms have a wide range of biological activities including antimicrobial, antioxidant, antiviral, anti-inflammatory, and other health promoting benefits [94,180,181,182,183,184]. Among the major bioactive ingredients of algae and mushrooms with demonstrated antimicrobial activities, proteins, antioxidants (polyphenols, flavonoids, and carotenoids), polyunsaturated fatty acids, and polysaccharides are the most important ones [185]. Until now, the antimicrobial potential of algae and mushrooms has been generally tested in vitro, providing reliable quantitative estimates of minimum inhibitory concentration (MIC) values for many samples [185,186]. Compounds reported to be present in algae included phlorotannins, terpenoids, phenolic compounds, acrylic acid, steroids, cyclic polysulphides, halogenated ketones and alkanes, and fatty acids that act as bactericidal agents [187]. The presence of these compounds suggests alternative mechanisms for antimicrobial action. For example, phlorotannins could inhibit the oxidative phosphorylation and bind with bacterial proteins including enzymes and cell membranes, leading to cell lysis [188]. The mechanisms of sulphated polysaccharides and algal polysaccharides may be related to glycoprotein receptors on the cell surface of polysaccharides which bind with compounds in the cell wall, cytoplasmic membrane, and DNA, increasing the cytoplasmic membrane permeability, protein leakage, and binding of bacterial DNA [189]. The antimicrobial activities of mushrooms may be related to a variety of secondary metabolites with biological activity, such as gallic acids, some phenols, volatile compounds, free fatty acids, and their derivatives [190]. Considering the wide biodiversity of mushrooms, they could easily become accessible sources of natural preservatives. However, few studies have evaluated the antimicrobial activities of algae and mushrooms in fish preservation. 

#### 3.2.5. Saponinse 

Saponins are natural glycosides compounds in some plants showing promising results as a broad-spectrum antimicrobial and antifungal activities [191,192]. The antifungal activity of saponins interacts with cytoplasmic membrane sterols, the ergosterol, can provoke pores and loss of membrane integrity, resulting in cell death [193]. 

#### 3.2.6. Flavonoids 

Flavonoids are ubiquitous in photosynthesizing cells and are commonly found in some plant parts. They exhibit broad-spectrum antimicrobial activities due to the ability to form complexes with extracellular and soluble proteins as well as with bacterial membranes [194,195,196]. Flavonoids have antimicrobial activities against bacteria and the hydroxyls at special sites on the aromatic rings of flavonoids improve the activity. However, the methylation of the active hydroxyl groups generally decreases the activity. The hydrophobic substituents such as prenyl groups, alkylamino chains, alkyl chains, and nitrogen or oxygen containing heterocyclic moieties usually enhance the activity for all the flavonoids [197]. As a whole, it is necessary to further investigate their potential use as food preservatives as people are increasingly interested in finding more natural alternatives.

### 3.3. Animal-Derived Compounds 

At present, many animal-derived antimicrobial compounds have also been used for fish preservation. Such examples include chitosan from shellfish, lactoperoxidase, and lactoferrin from milk, and lysozymes from hen eggs [198]. However, one of the major problems associated with animal-derived antimicrobials is their allergen risk; the sources of such ingredients are often allergen-containing foods including shellfish, milk, and egg [199]. 

#### 3.3.1. Chitosan 

Chitosan is a polycation biopolymer that naturally exists in the exoskeletons of arthropods and crustaceans [200]. Chitosan is the *N*-deacetylated form of chitin and linear polysaccharides with a variable degree of *N*-acetylation composed of more than 80 % β-(1,4)-2-amino-d-glucopyranose and less than 20 % β-(1,4)-2-acetamido-d-glucopyranose [201]. Chitosan and its derivatives have the characteristic of biodegradability, biocompatibility, bioadhesion, and nontoxicity, making it valuable compounds for medical, food, agricultural applications, and waste water treatment [30]. Chitosan has an excellent inhibitory effect on various microorganisms including some bacteria and fungi, and the antimicrobial action is intrinsically influenced by the type of chitosan, polymerization degree, natural nutrient constituency, host, nutrient or chemical composition of the substrates or both, and the environmental conditions (e.g., substrate water activity, moisture or both) [202,203,204]. Besides, chitosan has a novel application in the form of edible biopolymer-based films for bioactive compounds to extend shelf life of fish due to their abilities to retard oxygen, moisture, solute transports, and aromas [204]. More recently, some reviews have reported the application of chitosan as a bioactive film used in food including fish preservation [30,204,205,206,207,208,209,210]. 

#### 3.3.2. Lysozyme 

Lysozyme is an enzyme naturally found in mammalian milk and poultry eggs and is usually considered as a safety additive added directly to food [211]. It plays an important role in mediating protection against microbial invasion and the egg-white lysozyme is the most commercially available form of lysozyme [212]. Lysozyme could separate the β(1→4) bond between N-acetylglucosamine and N-acetyl-wall acid in the cell wall peptidoglycan of Gram-positive bacteria resulting in preventing them from invading. As reported by Rawdkuen et al., lysozyme has the highest antimicrobial activity against *S. cerevisia* and *Listeria*; however, it has no significant effect on Gram-negative bacteria because of the lipopolysaccharidic layer of outer membrane served as a physical barrier [213].

The outer membrane of Gram-negative bacteria could be destabilized by nisin or EDTA and the antimicrobial spectrum of lysozyme significantly increases [214]. Combination of 1000 IU/g of nisin and 160 μg/mL of oyster lysozyme treatment inside the calcium alginate coating could effectively retain the antimicrobial activity for 35 days [215]. Shi et al. used lysozyme to improve the quality of fresh pomfret preservation and lysozyme could inhibit the growth of Gram-positive bacteria and extended the shelf life by 1 to 2 days [216]. Wu et al. reported the utilization of chitosan-lysozyme coating could inhibit the microbial growth and lipid oxidation, maintain sensory qualities and extend the shelf life of refrigerated large yellow croaker [217]. The collagen-lysozyme active coatings could inhibit the microbial growth and decrease the total volatile basic nitrogen values on preserving fresh salmon fillets [218]. Besides, the lysozyme-enhanced bioactive films appeared to increase slightly in water vapor permeability [219]. 

#### 3.3.3. Lactoferrin

Lactoferrin is an 80 kDa whey glycoprotein consisting of two homologous globular lobes (N-lobe and C-lobe), and each of which binds an iron atom (Fe^2+^ or Fe^3+^) in synergy with the binding of one carbonate ion [220]. The antimicrobial activity of lactoferrin consists of two different and unrelated mechanisms, one on account of iron deprivation inhibiting the microbial growth, and the other associated with the large cationic patches presenting on the surface of lactoferrin [221]. Lactoferrin exhibits antimicrobial activity against a wide variety of bacteria and apolactoferrin was found to be bactericidal for *V. cholerae* and *S. mutans*, but not for *E. coli*. Further studies have demonstrated that lactoferrin has bactericidal effect only when in its iron-free state and that iron-saturated lactoferrin has a reduced antimicrobial activity [222]. Lactoferrin has been widely reported for its application as an adjuvant to improve cellular immune responses of fish [223,224,225]. However, few studies are available on its use in fish preservation.

#### 3.3.4. Lactoperoxidase

Lactoperoxidase is a member of the peroxidase family and the content of bovine milk is higher than that of other mammals’ milk [226]. Bovine lactoperoxidase is a glycoprotein consisting of a single peptide chain with a molecular weight of about 78.4 KDa and can catalyze the oxidation of thiocyanate ion (SCN-) in lactoperoxidase, generating oxidizing products such as hypothiocyanous acid (HOSCN) and hypothiocyanite (OSCN-). These products are known as antimicrobial agents that inhibit microbial growth by the oxidation of sulphydryl (SH) groups of microbial enzymes and other proteins [227,228,229]. Lactoperoxidase generally exhibits antimicrobial activity against Gram-negative bacteria, and the spoilage microorganisms of refrigerated fish have been considered to be caused by some Gram-negative bacteria, particularly *P. fluorescens* and *S. putrefaciens* [230]. Shokri et al. showed 1.25% (*v/v*) lactoperoxidase treated trout fillets could extend a shelf life by 4 days during storage; however, lipid oxidation in the fillets was not significantly affected by lactoperoxidase treatment [231]. Some studies used the active coatings incorporated with lactoperoxidase to improve the fish quality and extend the shelf life during storage [227,232,233]. At the same time, lactoperoxidase may also work in coordination with other natural preservatives or processing methods. For example, high hydrostatic pressure treatment at 450 MPa for 10 min combined with lactoperoxidase had good antimicrobial effects against *L. monocytogenes*, which could void biogenic amine formation for smoked salmon and prolong the shelf life [234]. In addition, Sharifi et al. indicated that lactoperoxidase combined with *Zataria multiflora* Boiss essential in alginate solution had a stronger effect on inhibiting the growths of *E. coli* O_157_: H_7_ and *L. monocytogene*s in trout fillets [235]. 

## 4. Conclusions

Microbial-, plant-, and animal-derived natural preservatives were summarized for fish preservation. Nisin, pediocins, reuterin, and lacticin as bacteriocins and organic acids and their sodium produced by microorganisms have been assessed in fish preservation and have shown antimicrobial activities against food-borne pathogens. The plant-derived compounds, including essential oils, plant extracts, and wood smoke, show excellent antimicrobial and/or antioxidant activities in fish preservation. Animal-derived antimicrobials including chitosan, lysozymes, lactoferrin, and lactoperoxidase have yet been evaluated and demonstrated to extend the shelf life of fish. In addition, the rich diversity of different algae and mushroom species can provide potential sources of new natural antimicrobial agents. It is evident that natural preservatives combined with either lower levels of synthetic/chemical ones, or with other hurdles, such as non-thermal sterilization processing, modified atmosphere packaging, and edible films and coatings, will enhance the performance of various natural preservatives discussed in the review.

## Figures and Tables

**Table 1:** Survey of literature dealing with bacteriocin employed biopreservation of fish.

Product	Bacteriocin Employed	Reported Effects	References
Skinless blue shark steak	Pediocin ACCEL	*L. monocytogenes*↓ ^1^	[67]
Oysters, mussels, clams	BacALP7, bacALP57	*L. monocytogene*s↓, *L. innocua*↓	[69]
Cold-smoked salmon	Divercin V41	*L. monocytogenes*↓	[78,79]
Fresh salmon fillets	Bacteriocin produced by *Lb. pentosus* 39	*A. hydrophila*↓, *L. monocytogenes* ↓	[80]
Reef cod	Enterocin CD1	The total viable count↓	[81]
Reef cod	Bacteriocin PSY2	The total count of spoilage bacteria↓	[82]
Pangasius fish fillets	Bacteriocin 7293	Target microorganisms↓	[83]
Reef cod filets	Bacteriocin GP1	Similar effect with that of sodium benzoate and the nisin B440	[84]
Fish pâté using fresh Nile tilapia	Bacteriocin produced by *L. lactis* 3MT	Vibrio↓	[85]
Fresh hake paste	Bacteriocin produced by *E. mundtii* STw38	Native flora of fish paste↓	[86]

^1^ Inhibited or decreased.

**Table 2:** Application of plant-derived natural preservatives in fish and other seafood.

Preservatives	Product Tested	Quality Attributes	References
Thyme essential oil	Minced silver carp fish	*L. monocytogenes* viable count↓ ^1^	[55]
Cinnamon oil	Northern snakehead fish fillets	Bacterial growth↓, TVB-N ^2^↓, thiobarbituric acid↓	[135]
Basil leaf essential oil	Sea bass slices	Total volatile base↓, peroxide value↓, TBARS ^3^↓	[136]
Carvacrol and thymol essential oil	Carp fillets	lipid oxidation↓, shelf life↑	[137]
Cinnamon oil	Rainbow trout	Microbial growth↓	[138]
Cinnamon, oregano and thyme essential oils	Salmon, scampi	Yeasts and molds↓, shelf life↑ ^4^	[139]
Clove essential oil	Sardine patties	Lipidic auto-oxidation↓, total mesophiles↓	[140]
Spearmint essential oil	Red drum fillets	Tissue hardness↑, protein degradation↓, nucleotide breakdown↓, microbiological properties↓	[141]
Rosemary essential oil	Silver carp	Lipid oxidation↓, total viable and psychrotrophic count↓	[142]
Horsemint essential oil	Bigheadcarp fillets	TVB-N↓, lipid oxidation↓, microbial deterioration↓	[143]
Oregano essential oil	Grass carp muscle	Total aerobic plate count↓, TVB-N↓	[144]
Oregano essential oil	Sea bream	TBARS↓	[145]
Oregano and thyme essential oils	Rainbow trout fillets	Shelf life↑	[146]
*Zataria multiflora* Boiss. essential oil	Rainbow trout fillets	TVB-N↓, total viable bacteria↓, lactic acid bacteria↓, *Pseudomonas* spp.↓	[147]
Tea polyphenol	Golden pomfret	Myofibril nanostructure↑, spoilage↓	[148]
Clove essential oil	Flounder fillets	Total volatile bases↓, pH↓, H_2_S-producing microorganisms↓	[149]
Clove essential oil	Bluefin tuna fillets	Microbial growth↓, lipid autooxidation↓	[150]
Cinnamon essential oil	Common carp	TVB-N↓, biogenic amines↓	[151]
Oregano essential oil	Fish fillets	Microbial growth↓, shelf life↑	[152]
Cinnamon bark oil	Grass carp fillets	Shelf life↑, *Aeromonas*, *Shewanella*, and *Pseudomonas*↓	[153]
Eucalyptus essential oil	Silver carp fillets	Total viable counts↓, total psychrotrophic counts↓, TVB-N↓, shelf life↑	[154]
Oregano essential oil	Grass carp	TBARS↓, TVB-N↓, putrescine↓, hypoxanthine↓, *Aeromonas* and *Shewanella*↓	[155]
Potato peel extract	Minced horse mackerel	Lipid and protein oxidation↓	[156]
Quince polyphenolic extract	Mackerel fillets	Fish oil oxidative deterioration↓, food-borne bacteria↓	[157]
Rosemary, sage tea extract	Sardine fillets	Histamine, putrescine and cadaverine accumulation↓	[158]
Grape seed and clove bud extracts	Silver carp fillets	Lipid and protein oxidation↓	[159]
Rosemary extract and onion juice	Sardine mince	Lipid oxidation↓	[160]
Rosemary extract	Crucian carp	TVB-N↓, *K*-value↓, TBARS↓	[161]
Grape polyphenols	Horse mackerel fillets	Lipid oxidation↓	[162]
Tea polyphenol, rosemary extract	Large yellow croaker	Maintained the good quality, shelf life↑	[163]
Tea polyphenol	Golden pomfret fish fillets	Troponin T degradation↓, spoilage VOCs ^5^↓, aerobic mesophilic/psychrotrophic count↓, yeasts and moulds↓	[148]
Tomato plant extract	Sierra fish fillets	ATP-related compounds↓, K value ↓, pH↓, total mesophilic count↓, shelf life↑	[164]
Pomegranate peel extract	Rainbow trout	Microbial growth↓, sensory and textural properties↑	[165]
Grape seed extract	Tilapia fillets	Trimethylamine↓, histidine↓	[166]
Pomegranate rind extract	Mackerel mince	Protein oxidation↓, carbonyl content↓, sulphydryl content↑, protein solubility↓	[167]
Shallot fruit and ajwain seed extracts	Rainbow trout fillets	Lipid oxidation↓, microbial spoilage↓, shelf life↑, sensory quality↑	[168]
Black cumin, black caraway extracts	Silver carp	Psychotropic bacteria↓, total viable counts↓, lipid oxidation↓	[169]
Pistachio green hull extract	Rainbow trout	Oxidative and hydrolytic rancidity↓, pH↓, TVB-N↓, histamine↓	[166]
Rosemary extract	Grass carp	Lipid oxidation↓, growth of bacteria↓, organoleptic quality↑, shelf life↑	[170]
Quinoa ethanolic extract	Atlantic chub mackerel	Lipid oxidation↓, lipid hydrolysis↓, pH↓, trimethylamine values↓	[171]
Grape seed extract	Tilapia fillets	Protein oxidation↓, maintained the morphology of myofibrils, freshness↑	[172]
Pomegranate peel extract	Nile tilapia fillets	Microbial counts↓, TVB-N↓, peroxide value↓, TBARS↓, sensory evaluation↑	[173]
Mint extract	Indian mackerel	Microbial proliferation↓, shelf life↑	[174]
*Allium paradoxum* and *Eryngium caucasicum* extracts	Silver carp fillets	Peroxide value↓, TBARS↓, acid value↓, TVB-N↓, bacterial growth↓	[175]
Fennel extract	Silver carp fillets	TVB-N↓, peroxide value↓, TBARS↓, microbial deterioration↓, shelf life↑	[176]
*Urtica dioica* L. extract	Rainbow trout fillets	Bacterial growth↓, TVB-N↓, TBARS↓	[177]
Pomegranate peel extract	Bighead carp fillets	Sensory quality↑, flesh color↑, spoilage bacteria↓, biogenic amines↓, ATP-related compounds↓, K-value↓	[178]
Summer savory extract	Spangled emperor fillets	Microbial growth↓, lipid oxidation↓, protein degradation↓, texture hardness↑, sensory properties↑	[179]

^1^ Inhibited or decreased; ^2^ Total volatile base nitrogen; ^3^ Thiobarbituric acid reactive substances; ^4^ Improved or increased; ^5^ Volatile organic chemicals.
